# Anti-Inflammatory Effect of Ethanolic Extract from *Tabebuia rosea* (Bertol.) DC., Quercetin, and Anti-Obesity Drugs in Adipose Tissue in Wistar Rats with Diet-Induced Obesity

**DOI:** 10.3390/molecules28093801

**Published:** 2023-04-28

**Authors:** Alejandro Barrios-Nolasco, Aarón Domínguez-López, Angel Miliar-García, Jorge Cornejo-Garrido, María Eugenia Jaramillo-Flores

**Affiliations:** 1 Laboratorio de Biología Celular y Productos Naturales, Escuela Nacional de Medicina y Homeopatía (ENMH), Instituto Politécnico Nacional, Guillermo Massieu Helguera 239, Col. La Escalera, Alcaldía Gustavo A. Madero, Ciudad de Mexico 07320, Mexico; barriosna84@gmail.com (A.B.-N.); jcornejog@ipn.mx (J.C.-G.); 2 Laboratorio de Biología Molecular, Escuela Superior de Medicina (ESM), Instituto Politécnico Nacional, Plan de San Luis y Díaz Mirón s/n, Col. Casco de Santo Tomas, Alcaldía Miguel Hidalgo, Ciudad de Mexico 11340, Mexico; aadominguezl@yahoo.com.mx (A.D.-L.); angel.miliar@yahoo.com.mx (A.M.-G.); 3 Laboratorio de Polímeros, Department de Ingeniería Bioquímica, Escuela Nacional de Ciencias Biológicas (ENCB), Instituto Politécnico Nacional, Wilfrido Massieu s/n esq. Manuel I. Stampa. Col. Unidad Profesional Adolfo López Mateos, Alcaldía Gustavo A. Madero, Ciudad de Mexico 07738, Mexico

**Keywords:** obesity, high-fat diet, adipose tissue, inflammation, mRNA, cytokines, *Tabebuia rosea*, quercetin, anti-obesity drugs

## Abstract

Obesity is characterized by the excessive accumulation of fat, which triggers a low-grade chronic inflammatory process. Currently, the search for compounds with anti-obesogenic effects that help reduce body weight, as well as associated comorbidities, continues. Among this group of compounds are plant extracts and flavonoids with a great diversity of action mechanisms associated with their beneficial effects, such as anti-inflammatory effects and/or as signaling molecules. In the bark of *Tabebuia rosea* tree, there are different classes of metabolites with anti-inflammatory properties, such as quercetin. Therefore, the present work studied the effect of the ethanolic extract of *T. rosea* and quercetin on the mRNA of inflammation markers in obesity compared to the drugs currently used. Total RNA was extracted from epididymal adipose tissue of high-fat diet-induced obese Wistar rats treated with orlistat, phentermine, *T. rosea* extract, and quercetin. The rats treated with *T. rosea* and quercetin showed 36 and 31% reductions in body weight compared to the obese control, and they likewise inhibited pro-inflammatory molecules: *Il6*, *Il1b*, *Il18*, *Lep*, *Hif1a*, and *Nfkb1* without modifying the expression of *Socs1* and *Socs3*. Additionally, only *T. rosea* overexpressed *Lipe*. Both *T. rosea* and quercetin led to a reduction in the expression of pro-inflammatory genes, modifying signaling pathways, which led to the regulation of the obesity-inflammation state.

## 1. Introduction

Obesity is a disease with an important inflammatory component that has complex interactions with our genes, diet, sedentary lifestyle, psychological–social influences, and epigenetic influences [1]. Obesity requires the identification of groups of molecules associated with the different subtypes of the disease for the design of precision medicine [2]. Examining the effect of currently available pharmacological options on the expression of target genes and comparing it with the effect of natural compounds provides insight into the potential of the latter [3]. The prevalence of obesity has been increasing in the last decade worldwide; by 2030, it is projected that one in five women and one in seven men will live with obesity (BMI ≥ 30 kg/m^2^), which is equivalent to over a billion people [4]. More than 95% of people with diabetes have type 2 diabetes, which is related to excess body weight. In 2014, 8.5% of adults older than 18 years had diabetes, and, by 2019 there was a 3% increase in diabetes mortality rates [5]. Regarding hypertension, it is currently estimated that 1.28 billion adults aged 30 to 79 years worldwide have hypertension [6], of which 78% in men and 65% in women are related to obesity [7]. Cardiovascular diseases (CVDs) are the leading cause of death worldwide [8]. An increase in the development of congestive heart failure is associated with between 31% and 40% of overweight people, compared with 32% and 49% of obese people [9]. Likewise, it is estimated that 17.9 million people died from CVDs in 2019, which represents 32% of all deaths in the world [8].

The genus *Tabebuia* is the largest of the *Bignonaceae* family. A wide variety of bioactive compounds are found distributed in each of the parts of the plant, with the bark being the most commonly used to obtain extracts rich in active compounds that include napthoquinones, quinines, furanonapthoquinones, benzoic acid, cyclopentenes, dialdehydes, and flavonoids [10]. *Tabebuia rosea*, also known as “ink trumpet tree”, is used in traditional medicine in some Latin American countries as an antimicrobial, astringent, diuretic, laxative, and to relieve symptoms such as fever, pain, and inflammation [11].

Quercetin is a flavonoid present in a variety of fruits, vegetables, and plants. It has a wide range of biological activities and health-promoting effects, including antioxidant, antidiabetic, anti-inflammatory, and anti-obesity properties [12]. Therefore, the consumption of foods rich in quercetin is beneficial to attenuate the adverse effects of hypercaloric diets. Quercetin improves the hypertrophy of adipose tissue by reducing the size of adipocytes [13] and decreases the accumulation of lipids in high-fat diet-induced obese rats [14].

Current options for pharmacological weight control include phentermine (indicated for short-term use only), and its role is focused on down-regulation of catecholamine concentration in satiety centers of the hypothalamus [15,16]. Orlistat (licensed for long-term use) acts by inhibiting gastric and pancreatic lipase and also functions as a ligand for the peroxisome proliferator-enabled receptor gamma (PPARγ), which is a sensor for lipase activity that is essential in the digestion of fat from the diet [17].

The inflammation associated with obesity is related to the accumulation of macrophages in the adipose tissue. Conventionally, inflammatory cytokines and chemokines are believed to be released by macrophages; however, hypertrophic adipocytes also produce them. The interactions between macrophages and adipocytes influence adipose tissue dysfunction by producing high levels of leptin, low levels of adiponectin, and systemic inflammation, favoring insulin resistance and increasing cardiovascular risk [18]. Hypoxia plays a fundamental role in the expansion of adipose tissue by inducing adipocytes to release pro-inflammatory factors such as TNF and IL6 [18]. Population studies link obesity to high levels of inflammatory cytokines such as IL1β, IL6, IL18, and TNF [19,20].

There are many molecules involved in the obesity-inflammation process, so the aim of this research was to evaluate the effect of the ethanolic extract from *Tabebuia rosea*, quercetin, and some drugs on the mRNA modulation of some obesity-inflammation associated genes in Wistar rats.

## 2. Results

### 2.1. Compounds Found in Tabebuia rosea Ethanolic Extract by UPLC Analysis

The mass spectrometry characterization of the *T. rosea* extract show that the compounds found in higher concentrations are Ursolic acid, Eriodictyol, Fisetin, Chamaemeloside, Salvigenin, Quercetin, Acanthopanolide B, Naringenin, 7-hydroxy-2-(1’ hydroxyethyl)naphto[2,3-b]furan-4,9-dione), 20R Ginsenoside, Harpagoside, and Khellin, among others. In lower concentrations, it is possible to detect the presence of two previously reported compounds, veratric acid and 5-hydroxy -2-(1’hydroxyethyl)naphto[2,3-b]furan-4,9-dione).

### 2.2. Anti-Obesogenic Effect in Wistar Rats

**Figure 1:**
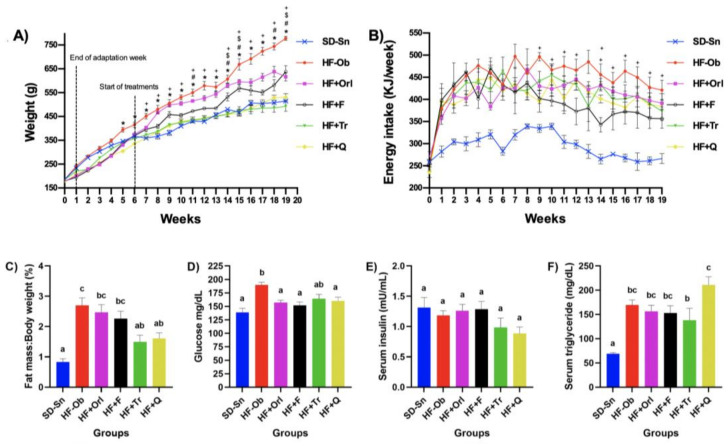
(**A**) Weekly body weight gain of rats; (**B**) Energy Intake; two-way analysis of variance (ANOVA) followed by a Bonferroni post hoc test. *p* < 0.05 was considered as significant. * Statistically significant difference between HF + Tr or HF + Q against HF-Ob. # Statistically significant difference between HF + Tr and HF + F. $ Statistically significant difference between HF + Q and HF + F. + Statistically significant difference between HF-Ob and HF + F. (**C**) Percentage of epididymal adipose tissue gained at the end of the experiment; (**D**) Serum glucose levels; (**E**) Serum insulin levels; (**F**) Serum triglyceride levels. Values are expressed as the mean ± SEM (*n* = 8). One-way analysis of variance (ANOVA) followed by a Bonferroni post hoc. *p* < 0.05 was considered significant. The different letters represent significant differences.

The healthy control group maintained on a standard diet without treatment (SD-Sn) show a weight of less than 33% with respect to the obese control group maintained on a hypercaloric diet without treatment (HF-Ob), while the obese group treated with orlistat (HF + Orl), phentermine (HF + F), *Tabebuia rosea* ethanolic extract (HF + Tr), and quercetin (HF + Q) show weight reductions of 20.7, 18.0, 36.6, and 31.7%, respectively, compared to the HF-Ob group. No statistically significant differences were observed among the SD-Sn, HF + Tr, and HF + Q groups (Figure 1A), while Figure 1B shows that the groups with the lowest energy consumption were those treated with the extract and phentermine, still distant from SD-Sn, and those with the highest consumption, the HF-Ob group. Figure 1C shows the amount of epididymal adipose tissue in regard to the total body weight expressed as a percentage of the experimental groups, where the highest amounts were obtained as follows: in the HF-Ob, HF + Orl, and HF + F groups compared to the SD-Sn control group, while the HF + Tr and HF + Q groups had less adipose tissue gain, both show a significant difference with respect to the HF-Ob group.

### 2.3. Glucose and Insulin Levels and Lipid Profile Analyses

In glucose metabolism, a significant increase in serum glucose values in the HF-Ob group (189.8 ± 4.9) was observed compared to the SD-Sn group (138.7 ± 7.8), while the HF + Q and HF + Tr groups did not show a difference with respect to SD-Sn (Figure 1D). While serum insulin levels did not present changes in all groups (Figure 1E).

In the analysis of the lipid profile, a significant increase in triglycerides (Figure 1F), total cholesterol, and VLDL could be observed in the groups that received a high-fat diet. In the case of HDL, an increase was observed in all groups except HF-Tr, while the LDL values of the HF-F and HF-Q groups show a decrease compared to the HF-Ob group (Table 1).

### 2.4. Adipose Tissue Morphology

**Figure 2:**
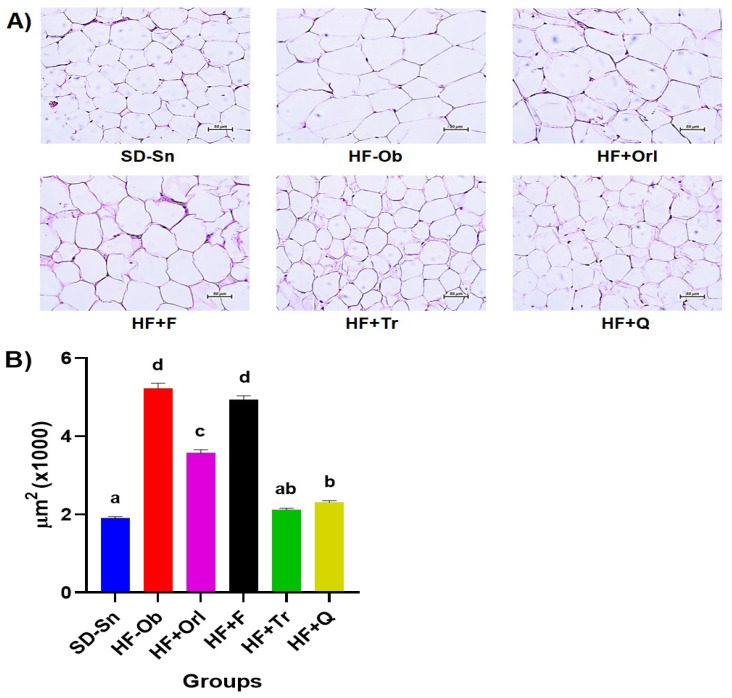
(**A**) Representative micrographs of adipose tissue sections from the experimental groups stained with hematoxylin and eosin (H&E), observations at 20×. (**B**) Comparison of adipocyte size. Values are expressed as the mean ± SEM (*n* = 400). One-way analysis of variance (ANOVA) followed by a Bonferroni post hoc test. *p* < 0.05 was considered significant. The different letters represent significant differences.

Figure 2A shows the micrographs of adipose tissue sections from the different groups. ImageJ software was used to analyze adipose tissue based on adipocyte size. Figure 2B shows that the animals treated with the extracts of *T. rosea* and quercetin reduced the size of the adipocytes (2115 ± 42 and 2307 ± 47 μm^2^, respectively) to the same level as the healthy group (SD-Sn, 1904 ± 44 μm^2^), with the HF + Q group being statistically different from the healthy control group. Likewise, the rats with the largest adipocytes (5227 ± 129 μm^2^) were observed in the control group HF-Ob, followed by the HF + F (4932 ± 105 μm^2^) and HF + Orl (3567 ± 89 μm^2^), the latter being statistically different from the HF-Ob group.

### 2.5. Effect of Ethanolic Extract from Tabebuia rosea, Quercetin, and Anti-Obesity Drugs on mRNA Expression of Pro-Inflammatory and Anti-Inflammatory Cytokines

**Figure 3:**
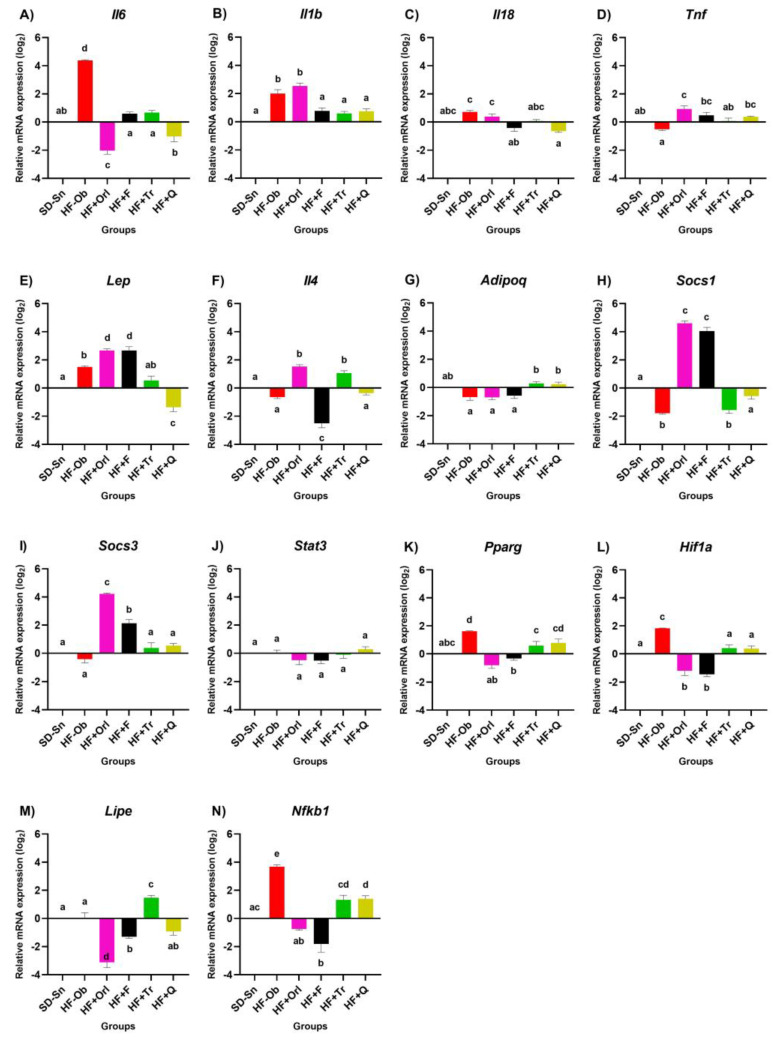
Effect of the extract of *Tabebuia rosea,* quercetin, and anti-obesity drugs in obese Wistar rats on pro-inflammatory (**A**–**E**) and anti-inflammatory cytokines (**F**,**G**); cytokine signaling suppressors (**H**,**I**); transcription factors (**J**–**N**), and *Lipe*. Values expressed as the mean ± error std. (*n* = 8). One-way analysis of variance (ANOVA) followed by a Bonferroni post hoc test. *p* < 0.05 was considered significant. The different letters represent significant differences.

The effect of the different treatments on the obese animals is shown in Figure 3A. An increase in *Il6* mRNA levels was observed in the HF-Ob group, while the rest of the treatments reduced the expression to normal levels. In Figure 3B, obese animals without treatment (HF-Ob) show a significant increase in *Il1b* compared to the SD-Sn group, but the HF + Orl group shows the highest-level expression. The groups HF + Tr, HF + Q, and HF + F reduced the levels of this cytokine almost to the levels of the SD-Sn group (healthy). The *Il18* results (Figure 3C) show no differences between the SD-Sn group and the HF-Ob group, while the HF-F and HF-Q treatments decreased mRNA expression levels. In Figure 3D, it was observed that the *Tnf* mRNA expression level of the SD-Sn and HF-Ob groups is very similar, with no significant difference between them. The treatment with orlistat shows the highest level of expression with respect to all the experimental groups, statistically higher only against the HF-Ob. The effect of treatments on *Lep* mRNA expression is shown in Figure 3E, where orlistat and phentermine treatments show significantly higher mRNA levels than all groups. On the other hand, the group HF + Q shows a statistically lower level of mRNA expression compared to all groups, except against the SD-Sn group. Regarding *Il4*, no significant differences were observed between the HF-Ob and SD-Sn control groups (Figure 3F); however, the animals treated with the extract (HF + Tr) and orlistat show a significantly higher increase in this anti-inflammatory cytokine compared to all the experimental groups. In the case of *Adipoq* (Figure 3G), no differences were found in the expression levels between the groups against HF-Ob, with the exception of HF + Tr, which increased them. In Figure 3H, the HF + Orl and HF + F groups increased *Socs1* mRNA expression, as they are statistically different from each other. The treatments HF + Tr and HF + Q did not present statistically significant differences between them. For *Socs3* (Figure 3I), a similar effect to *Socs1* was observed. Regarding *Stat3*, no differences were found between HF-Ob and SD-Sn controls. However, quercetin significantly increased *Stat3* mRNA levels (Figure 3J). For *Pparg*, all treatments significantly reduced expression levels relative to the HF-Ob group; HF + Orl and HF + F were the treatments that reduced the expression levels of this mRNA to a greater extent, which in turn were lower compared to the HF + Tr and HF + Q groups (Figure 3K). For *Hif1a*, all treatments significantly reduced expression levels with respect to the HF-Ob group; the HF + Orl and HF + F treatments reduced this mRNA to the highest level (Figure 3L). For *Lipe* (Figure 3M), no differences were observed between the SD-Sn and HF-Ob groups; an overexpression was observed in the HF + Tr group against the rest of the experimental groups. As for the groups HF + Orl, HF + F, and HF + Q, no significant difference was observed between them; these last groups had the lowest level of expression of *Lipe*. In the case of the transcription factor *Nfkb1* (Figure 3N), an overexpression was observed in the obese control group compared to all the experimental groups, which strongly reduced the expression, with no significant difference between them.

## 3. Discussion

Several studies have focused on the anti-inflammatory effect of different extracts obtained from the leaves, stems, roots, and barks of different plant species, contributing to the discovery of a considerable number of molecules in recent decades. In this research, we identified the main compounds present in the extract of *T. rosea*, and their effects on the expression of different inflammatory biomarkers. Both the extract and quercetin could have the ability to specifically inhibit the mediators of the inflammatory response.

The group of rats treated with the ethanolic extract of *T. rosea* achieved the lowest weight gain among the five groups during the 13 weeks of treatment. The weight of the rats that were fed HF was reversed by the administration of *T. rosea* ethanolic extract as well as quercetin. This result is consistent with others in which quercetin consumption could effectively reduce body weight in hypercaloric-fed rats [21].

Quercetin improves glucose levels by attenuating oxidative stress and increasing antioxidant capacity [22], while the ethanolic extract of *T. rosea* decreases glucose values in obese C57BL/6 mice, possibly due to the overexpression of GLUT-4 in adipocytes [23]. Quercetin (10 mg/kg for 10 weeks) decreases obesity, insulin resistance, dyslipidemia, and hypertension in obese Zucker rats [24], and, at a dose of 50 mg/kg intragastrically for 15 days, it may serve as a novel therapeutic approach to prevent obesity-mediated liver damage [25].

Obesity produced by a high-fat diet can produce hepatic steatosis, which causes an increase in transaminase, LDL, VLDL, and triglyceride levels [26]. In accordance with this, our results show an increase in these values. The increase in HDL levels could be associated with the increase in ApoA-I and ApoA-II due to the hypercaloric diet administered to obese rats (obviously, this needs to be verified experimentally). This increase is directly related to the levels of ApoA-II in mice. The Apo-II gene encodes high-density lipoproteins (HDL), as well as a gene called Ath-1, which controls HDL levels in response to a diet high in fat and cholesterol [27].

Accumulating evidence has indicated that quercetin could protect against obesity and its associated metabolic syndrome through different molecular pathways. In rat adipocytes, quercetin can inhibit the uptake of glucose by directly binding to the glucose transporter GLUT4. Quercetin can also reduce obesity in high-fat diet-fed rats by modulating the gut microbiota. The in vitro inhibition of quercetin on pancreatic lipase was also investigated in some studies. However, the in vivo effects of quercetin on fat absorption and excretion are being investigated ([28]).

Adipose tissue triglyceride lipase (ATGL) is the key enzyme for the release of fatty acids from triacylglycerol stores. Both intermediate and final lipolytic breakdown products also have critical regulatory roles affecting cell signaling, gene expression, metabolism, cell growth, cell death, and lipotoxicity. Therefore, the regulation of ATGL is vital to maintaining a defined balance between lipid storage and mobilization.

ATGL plays a prominent role in this reaction by catalyzing the initial step of cleaving TG to diacylglycerol (DG) and FA. Immediately, hormone-sensitive lipase (HSL) hydrolyzes DG to monoacylglycerol (MG) and FA, whereas monoglyceride lipase (MGL) cleaves MG to glycerol and FA. As the tight regulation of lipolysis is of great physiological importance, ATGL is regulated on different levels. Transcriptional control of ATGL is extensively reviewed elsewhere. Post-transcriptionally, ATGL is regulated by different proteins, which work in concert to ensure ATGL’s proper function. Human patients with a loss of functional ATGL suffer from a neutral lipid storage disease characterized by abnormal TG accumulation in multiple organs and tissues, leading to cardiac and skeletal muscle myopathy. Besides massive TG accumulation, global inactivation of ATGL in mice results in reduced FA concentrations in the plasma, having beneficial effects on glucose tolerance and insulin sensitivity [29]. Various reports show the inhibition of different types of lipases [30]; therefore, if quercetin is exerting inhibitory effects on ATGL, this explains the accumulation of TG in serum that is observed, which obviously must be verified.

Our results also show a decrease in the *Lipe* transcript, which could mean that the activity of the protein is decreased and, therefore, there is no hydrolysis of triglycerides, and consequently, their concentration increases.

In obesity, a low-grade inflammatory state persists, induced by lipotoxicity generated by excess energy from the diet. It is widely documented that the pro-inflammatory cytokines TNF-α, IL-6, IL-1β, and IL-18 are overexpressed in obese individuals. TNF-α, whose levels are reduced in healthy individuals, is secreted by macrophages resident in adipose tissue, is a very potent pro-inflammatory cytokine, and is secreted first by myeloid cells via activation of the MAPK and NFκB signaling pathways, whose overexpression is correlated with insulin resistance [31].

*Il6* mRNA expression in the adipose tissue of high-fat diet-induced obese animals has been reported to be significantly elevated [32]. Elevated levels of IL-1β, IL-6, and TNF-α impair systemic insulin sensitivity and promote cancer development [33,34]. This increase in *Il6* mRNA expression observed in the HF-Ob group may be associated with the activation of *Nfkb1* in a pro-inflammatory environment by IL-6, which in turn promotes an inflammatory response and induces the expression and secretion of cytokines and chemokines, such as IL-6 and IL-1β [35]. Therefore, the overexpression of *Il1b* in the HF-Ob group could occur due to the increase in *Hif1a*, despite the fact that in the obese control group, *Stat3* was not overexpressed compared to the other treatments, but *Il6* was. The ethanolic extract of the bark *Tabebuia impetiginosa* inhibits the production of IL-1β and IL-6 in human peripheral blood mononuclear cells (PBMCs) activated by phorbol myristate acetate (PMA)/ionomycin [36], which agrees with the results observed for the ethanolic extract of *T. rosea.* An increase in *Il1b* mRNA expression was observed in orlistat-treated animals compared to SD-Sn and HF-Ob, a result similar to that reported in orlistat-treated high-fat diet obese mice [37]. Flavonoids, such as apigenin and quercetin, act by inhibiting the activation of the JAK-STAT pathway in lymphocytes [38] and chondrocytes [39]. Other studies have shown that flavonoids can directly bind to some protein kinases to inhibit them, which include Janus kinase 1 (JAK1), phosphoinositide 3-kinase (PI3K), and mitogen-activated protein (MAP). In our research, the decrease in *Il6*, *Il1b*, and *Il18* mRNA in the treatment with quercetin could have occurred through the inactivation of the JAK-STAT pathway, specifically binding to the JAK1 protein and not to STAT, similar to *T. rosea* since the extract is rich in polyphenols. The decrease in the expression of *Il6* with orlistat is similar to that reported by other researchers in liver tissue [40] and in adipose tissue [41].

*Il4* promotes the expression of *Pparg* and induces the differentiation of M2 macrophages, which are essential for the development of functional beige fat. Regarding the administration of quercetin, there were no changes in the levels of *Il4* with respect to the HF-Ob group, unlike the reports that indicate that the administration of this compound decreases the expression of this cytokine [42,43]. In contrast, the *T. rosea* extract increased the expression of *Il4*, promoting the anti-inflammatory state, which can be explained by the presence of ursolic acid in the extract, which exhibits anti-inflammatory activities [44].

Several cytokines, including IL-1β, IL-6, IL-18, and TNF-α, play a role in modulating food intake through the central nervous system [45,46,47,48]. Although no difference was found between the HF-Ob group and the SD-Sn group, the treatment with orlistat and quercetin induced the overexpression of *Tnf* mRNA. This result is similar to that reported by Y. Xu et al. in 2015 [41], where they observed an increase in the expression of this gene treated with polysaccharides, polyphenols, and caffeine compared to the healthy control group. The expression of *Tnf* mRNA induced by *T. rosea* and quercetin could indicate the beginning of an anorexic effect, such as TNF-α, which reduces food intake in rats by a direct action on the central nervous system [48]. It is likely that to find high levels of *Tnf* in obese animals, longer times of ingestion of a high-fat diet are required. Research carried out with high-fat diet-induced obese CD-1 mice showed that they only achieved a significant difference in adipose tissue for *Tnf* mRNA after 12 weeks [49].

Within adipose tissue, *Il18* mRNA is specifically expressed in vascular stromal cells [50]. IL-18 has been shown to be involved in chronic low-grade inflammation that may be a pathogenic factor behind metabolic syndrome and type 2 diabetes mellitus [51]. The expression of *Il18* shows significant differences between the SD-Sn and HF-Ob groups. *Il1b* and *Il18* lead to an inflammatory state, as observed in this study, since *Il1b*, *Il18*, *Il6*, *Lep*, and *Hif1a* were found to be overexpressed in the HF-Ob group, a pro-inflammatory process that is predominantly activated by IL-1β, and the treatments induced the downregulation of all of these. The plasma *Lep* concentration and mRNA expression in adipose tissue are directly related to the severity of obesity since an increase in fat mass is associated with an increase in *Lep*, making leptin an indicator of total fat mass [52]. In this study, orlistat and phentermine increased the levels of *Lep* mRNA, such that if protein expression is consistent with the messenger, leptin binds to its receptor, activating the formation of the ObRb/JAK2 complex to activate STAT3, forming the activated complex that translocates to the nucleus to bind to DNA, and inducing the expression of *Socs3*, which inhibits the same pathway by negative feedback; therefore, it would be expected that high levels of leptin produce high levels of *Socs3*, which would eventually induce a reduction in leptin and lower levels of *Socs3*. This signaling pathway was altered, as when *Lep* mRNA expression levels increased, it resembled increases in *Socs3* expression, but there was a downregulation of *Stat3* (compared to the obese group) in the case of the drugs, which would eventually lead to a reduction in *Socs3* levels and maintain high *Lep* mRNA levels. In contrast, in the case of quercetin, the leptin levels were reduced, possibly due to a prolonged activation of STAT3, whose expression was increased, without modifying the levels of *Socs3*.

Orlistat treatment reduced body weight, adipose tissues, and serum lipids compared to hypercaloric-fed rats. Orlistat is a reversible lipase inhibitor that works by inhibiting the absorption of dietary fats. Consequently, with decreased body weight and total adipose tissue levels, the serum level of inflammatory markers was significantly reduced. Furthermore, serum LEP decreased in response to orlistat treatment [53]. SOCS3 is a negative regulator of *Il6*, and *Socs3* deficiency induces the prolonged activation of *Stat3*, as is clearly observed with *T. rosea* and quercetin. *Socs3* did not show changes in the HF-Ob group, and a slight change can be observed in the treatments with *T. rosea* extract and quercetin.

Several recent studies have shown that adipocyte *Adipoq* gene expression is negatively regulated by TNF-α secreted by macrophages recruited to the stromal vascular fractions contained in hypertrophic adipose tissue [54]. A non-significant increase in mRNA levels of *Adipoq* was observed in all treatments compared to the HF-Ob group but is very similar to the SD-Sn group. However, *Adipoq* expression levels are known to be reduced in rats with diet-induced obesity [55], which was increased with HF-Tr, and a reduction in *Lep* levels was also observed in the treatment with quercetin. Other studies carried out with Welsh onion ethanolic extracts in obese mice show an increase in the levels of *Adipoq* mRNA compared to the obese control group [56].

As a countervailing effect to those of the inflammatory process, the extract of *T. rosea* increased levels of *Il4* and *Adipoq*, and consequently improved the sensitivity to insulin. The decrease in *Pparg* levels is consistent with the fact that quercetin can decrease the levels of *Pparg* in 3T3-L1 adipocyte cells, and what was observed with the mulberry leaf polyphenol extract, which inhibits preadipocyte differentiation and hepatic lipogenesis with the suppression of *Pparg* [57]. The same results were observed with Kaempferol administered to obese C57BL/6 mice [58] and with the ethanolic extract of *T. avellanedae* [59]. An increase in *Pparg* is observed in the HF-Ob control group compared to the SD-Sn control group; however, all the treatments decrease the expression of *Pparg* compared to HF-Ob. A decrease in *Pparg* mRNA was observed in animals treated with orlistat compared to the obese control group (HF-Ob). Similar results are observed in studies on mice with diet-induced obesity, where there is also a decrease in *Pparg* mRNA in epididymal adipose tissue [56,60].

The extract of *T. rosea* induced the overexpression of lipase E (*Lipe*), indicating that this is one of its main mechanisms for weight reduction. The expression of genes encoding lipases (*Pnpla2*, *Lipe*, and *Mgll*) is decreased in obese individuals [61], which is consistent with our results (Figure 3N, HF-Ob group), while orlistat has been shown in in vitro activity assays to inhibit ATGL and LIPE [62]. The suppression in the expression of LIPE in treatments with orlistat is due to the fact that this compound is a selective inhibitor of this molecule [63]. Orlistat is a well-known pancreatic lipase inhibitor that also inhibits HSL (hormone-sensitive lipase), thereby inhibiting stimulated lipolysis [64,65].

A significant up-regulation of *Nfkb1* mRNA was observed in the group treated with orlistat compared to the obese control group, unlike what was reported (downregulation) by Wu et al. in 2016 [37] in a mouse model with colon cancer, suggesting that orlistat may ameliorate inflammatory effects through the inhibition of *Nfkb1* and *Stat3* [66].

ADIPOQ has anti-inflammatory functions, and high consumption in diets rich in saturated fat reduces its expression and concentration in the obese state [67]. Likewise, ADIPOQ is related to energy homeostasis, immune functions, and vascular regulation [68]. In cultured 3T3-L1 adipocytes, acriflavin inhibits HIF1α, induces ADIPOQ expression, and reduces SOCS3 expression [69]. The ethanolic extract of *T. rosea* inhibited the expression of *Hif1a* when compared to the obese group. *T. rosea* did not induce or inhibit the expression of *Socs 1* and *3*, which function as inhibitors of cytokine signaling; therefore, the signaling pathway for the inhibition of cytokines must be through the induction of other molecules, such as protein A20 [70].

In rodent adipose tissue, it has been reported that *Socs3* overexpression appears to occur in response to TNF-α release, and TNF-α deficiency causes low levels of *Socs3* [71,72]. The overexpression of *Socs* genes in treatments with anti-obesity drugs may be related to *Lep* overexpression since it is reported that central resistance to leptin may involve the leptin suppressor. The SOCS3 cytokine signaling family is induced by leptin and prevents activation of the JAK-STAT pathway [73]. This would explain, in part, the under-expression of proinflammatory cytokines observed in this study. SOCS proteins inhibit signaling either by direct inhibition of JAK (SOCS1) kinase activity or recruitment of SH2 into the cytoplasmic domain of the receptor, followed by inhibition of JAK (SOCS3) activity [74,75].

Obesity is the result of the imbalance between high caloric intake and lack of energy expenditure, which leads to the constant expansion of adipose tissue, giving rise to hypertrophy and hyperplasia and causing greater permeability of adipose tissue to macrophages, where the lack of oxygen results in the activation of HIF-1α, which in turn is responsible for the activation of macrophages and the production of proinflammatory interleukins, giving way to low-grade chronic inflammation. In the obese animal, it can be observed that an increase in the expression of HIF-1α, which induces the overexpression of proinflammatory cytokines in addition to LEP, exacerbates the inflammatory state. It is also important to note that the upregulation of *Tnf* reduces the expression of *Socs1*. Likewise, the overexpression of proinflammatory cytokines downregulates *Socs1* and *Socs3*, which leads to the activation of STAT3. In the case of proinflammatory cytokines, the downregulation of proinflammatory cytokines except for *Tnf* is observed, reducing the expression of *Socs* and increasing *Stat* as mentioned above.

Another aspect to consider in obese people is the composition of the microbiome, which plays a very important role in metabolic alterations, anti-inflammatory processes, and the integrity of the intestinal mucosa, among others. Short-chain fatty acids (acetate, propionate, formate, butyrate, lactate, and succinate) (SCFA) are produced when soluble dietary fiber and resistant starch are fermented by intestinal microorganisms in the colon.

These SCFA are absorbed and promote lipogenesis through their activating effects on transcription factors CHREBP and SREBP1 as well as the inhibition of lipoprotein lipase, leading to the accumulation of triglycerides in host adipocytes. Therefore, SCFA synthesis by the gut microbiome may trigger triglyceride accumulation in host adipocytes through various regulatory mechanisms. Another proposed mechanism is that the gut microbiome decreases the oxidation of fatty acids in the liver due to the inhibitory effect on adenosine monophosphate kinase (AMPK).

The altered B/F ratio reduces the proteins that make up the junctions, resulting in a possible translocation of lipopolysaccharides (LPS), which constitutes one of the first stages that trigger the proinflammatory cascade. In a high-fat diet as well as an LPS-supplemented diet, binding to TLR4 can occur, initiating the proinflammatory cytokine cascade, but only in the presence of CD14. Conversely, if CD14 is not present, the inflammatory response never starts. The same occurs when TLR4 is not expressed [76].

## 4. Materials and Methods

### 4.1. Plant Material and Preparation of the Ethanolic Extract

The outer bark of *T. rosea* was collected in the State of Campeche, Mexico, in September 2018 and was taxonomically identified in the Herbarium of the Facultad de Estudios Superiores-Iztacala (FESI) of the Universidad Nacional Autónoma de México (UNAM). One specimen was deposited in the herbarium and assigned a voucher number of 2428.

Previously crushed bark (1 kg) was placed into 6.5 L of ethanol for 5 days, and this extraction process was repeated three times at room temperature (25 °C). Subsequently, the extract was filtered and concentrated to dryness in a Rotavapor (Büchi R-215, Flawil, Switzerland), coupled to a V-700 vacuum pump and a F-105 recirculator (Büchi, Flawil, Switzerland). The dry extract was stored in amber bottles at −20 °C until use.

### 4.2. Ultra Performance Liquid Chromatography (UPLC) Analysis

The mass spectrometry analysis was carried out in an Acquity Ultra Performance Liquid Chromatography I-class system with a diode array detector (Waters Corp., Milford, MA, USA) coupled to a mass spectrometer with an ESI ionization source and time of flight VION IMS (Waters Corp., Milford, MA, USA). The analysis conditions are presented in the supplementary material.

For data analysis, UNIFI, version 1.9 SR4 Software (Waters Corp., Milford, MA, USA), was used with the libraries of the Specialized Food Analysis Laboratory, the University of Mississippi Botanical Library, and the University of Ottawa Phytochemical Library. Target match tolerance set to 5 ppm. For the identification of fragments, it was compared with fragmentation patterns reported in PubChem, FooDB version 1.0, and HMDB version 5.0.

### 4.3. Animals

Forty-eight male Wistar rats with weights of 180 ± 20 g were acquired from the bioterium of the Facultad de Estudios Superiores de Iztacala of the Universidad Nacional Autónoma de México, México. The animals were handled according to the Code of Ethics for Animal Studies of the Escuela Nacional de Medicina y Homeopatía (Reg. num. CBE/005/2021) and the Guide for the Care and Use of Laboratory Animals of the Mexican Council for the Care of Animals (NOM-062-ZOO-1999), complying with the International Standards and Policies on the use, care, and humane slaughter of animals from laboratories.

### 4.4. High-Fat Diet-Induced Obese Wistar Rats Treatments

The animals were kept in acrylic cages with sterilized sanitary beds under standard conditions at 22–23 °C with photoperiods of 12 h light/12 h dark and access to food and water *ad libitum*. During a week of adaptation, all the animals consumed a standard diet (SD) 5001 Labdiet^@^ Rodent Laboratory Chow, (Land O´Lakes, Inc, Arden Hills, MN, USA) with an energy density of 3.2 kcal/g; the formulation of the standard diet is shown in Table S1. Later, they were randomly divided into six groups (*n* = 8), according to the treatment to be evaluated. One group of animals was fed SD during the entire experiment, which was identified as SD-Sn (healthy control, without treatment); then, five groups received a high-fat diet (HF) for 5 weeks to promote the development of the obesogenic phenotype, reaching a weight of 350 ± 20 g. The high-fat diet had an energy density of 4.5 kcal/g and was prepared in our laboratory with the ingredients shown in Table S1 [77]. Obese animals were maintained on a HF diet throughout the experiment, and the following treatments were administered intragastrically once a day (100 µL/100g body weight) for 13 weeks: HF-Ob (obese control, untreated), HF + Orl (obese group treated with Orlistat, 5.14 mg/kg, Liomont Laboratories), HF + F (Phentermine, 10 mg/Kg, ifa CELTICS), HF + Tr (ethanolic extract of *T. rosea*, 150 mg/Kg) and HF + Q (Quercetin, 10 mg/Kg, Sigma-Aldrich Q4951, St Louis, MO, USA). All treatments were prepared in Dimethyl sulfoxide (DMSO, Sigma-Aldrich D4540, St Louis, MO, USA) in water USP (1:3) (0.35 mg/kg of DMSO once a day).

### 4.5. Biochemical and Histopathological Analysis

At the end of the treatments, the animals were sacrificed by cardiac puncture after being anesthetized with a mixture of xylazine (5 mg/kg) and ketamine (40 mg/kg). The biochemical analysis for the determination of glucose, insulin, cholesterol, and triglyceride levels was carried out in an AutoKem-II system (KontroLab, Rome, Italy). Finally, epididymal adipose tissue samples were preserved in RNAlater^TM^ (Sigma-Aldrich R0901, St. Louis, MO, USA) in cryogenic tubes and stored at −85 °C until use. One section of adipose tissue was fixed in 10% formalin-PBS, embedded in paraffin, and cut into 5 μm slices that were stained with hematoxylin and eosin for histopathological analysis under microscopy (20×). The Image J version 1.53t software (National Institutes of Health, Bethesda, MD, USA) with the adipocytes tool was used to quantify adipocyte size.

### 4.6. RNA Extraction

The extraction and purification of RNA from adipose tissue were carried out using the E.Z.N.A Total RNA Kit II (Omega, Bio-tek, Inc., Norcross, GA, USA) following the manufacturer’s recommendations with 200 mg of adipose tissue, adding 20 μL of 2-mercaptoethanol (Sigma-Aldrich M6250, St Louis, MO, USA) to inhibit DNases and RNases. A BeadBug homogenizer (Benchmark Scientific, Inc., Sayreville, NJ, USA) was used to disintegrate the tissue into BeadBug tubes with 1 mm zirconium beads (Sigma-Aldrich Z763780, St. Louis, MO, USA). RNA concentration (OD-260) and purity (OD-260/OD-280) were determined by depositing 1 µL of RNA on the UV-Vis NanoDrop spectrophotometer (Thermo Scientific ND1000, Wilmington, DE, USA).

To verify the integrity of the RNA, 1% agarose (BioRad 1613102, Hercules, CA, USA) gel electrophoresis was performed using a BioRad horizontal electrophoresis system (BioRad 1704467, Hercules, CA, USA).

### 4.7. Real-Time PCR (qPCR)

The reaction was carried out with SuperScrip II Reverse Transcriptase (Invitrogen, 18064014, Waltham, MA, USA) for the synthesis of cDNA. Finally, the cDNA was subjected to qPCR using the LightCycler FastStar DNA Master SYBR Green Ι kit (Roche 03003230001, Mannheim, Germany) with 0.5 µM concentrations of oligonucleotides corresponding to the mRNAs of the anti-inflammatory genes *Il4* and *Adipoq*; pro-inflammatory genes *Il6*, *Il1b*, *Il18*, *Tnf* and *Lep*; transcription factors *Socs1*, *Socs3*, *Stat3*, *Pparg*, *Hif1a* and *Nfkb1*; oligonucleotides of the lipase E (*Lipe*); and *B-actin* as the reference gene. All of the oligos were designed with the Primer 3 Web Tool (http://bioinfo.ut.ee/primer3–0.4.0/primer3) (Table 2). The PCR conditions were as follows: 95 °C for 10 min, 40 cycles at 95 °C for 10 s, 60 °C for 10 s and 72 °C for 10 s. Relative gene expression was calculated by the ΔΔCt method using expression data of the *B-actin* gene as the normalizer.

### 4.8. Statistical Analysis

A Shapiro–Wilk normality test was performed on all data sets, and *p* values > 0.05 were considered normal. For this test, the IBM SPSS Statistics software, Version 29.0 (Armonk, NY, USA: IBM Corp.) was used. Subsequently, the data sets were analyzed using one-way analysis of variance (ANOVA) and a Bonferroni post hoc test to evaluate the differences between each experimental group with a 95% confidence interval. It was verified that there was no significant difference in the variance of the groups with a Brown-Forsythe test (*p* > 0.05). These analyses were carried out in GraphPad Prism version 6.0 software (GraphPad Software, San Diego, CA, USA).

## 5. Conclusions

The ethanolic extract of *Tabebuia rosea* and quercetin inhibited the molecules associated with pro-inflammatory states *Il6*, *Il1b*, *Il18*, *Lep*, *Hif1a*, and *Nfkb1*, without modifying the expression of *Socs1* and *Socs3*. In addition, the *T. rosea* extract increased the expression of *Lipe*. Both extracts of *T. rosea* and quercetin led to a reduction in the expression of pro-inflammatory genes and the modification of signaling pathways, which led to the regulation of the obesity-inflammation state. Therefore, the extract of *T. rosea* or quercetin could be used for the development of new drugs for the treatment of obesity. However, future experiments are necessary to know the protein levels and to be able to confirm the regulation of expression at the transcriptional level.

## Figures and Tables

**Table 1:** Serum lipid profile of the experimental groups.

	SD-Sn	HF-Ob	HF + Orl	HF + F	HF + Tr	HF + Q
Total Cholesterol (mg/dL)	53.2 ± 2.7 ^a^	77.0 ± 4.3 ^b^	86.3 ± 3.8 ^b^	84.4 ± 3.6 ^b^	81.2 ± 2.0 ^b^	82.0 ± 3.1 ^b^
HDL (mg/dL)	17.4 ± 0.7 ^a^	15.8 ± 0.7 ^a^	26.8 ± 1.5 ^b^	24.6 ± 2.4 ^b^	16.8 ± 0.7 ^a^	27.0 ± 0.9 ^b^
LDL (mg/dL)	24.5 ± 3.4 ^ab^	30.6 ± 3.8 ^c^	26.0 ± 3.0 ^bc^	18.2 ± 1.7 ^a^	35.7 ± 4.8 ^c^	19.9 ± 2.5 ^a^
VLDL (mg/dL)	15.5 ± 1.3 ^a^	30.7 ± 2.0 ^b^	32.3 ± 2.5 ^b^	34.4 ± 1.6 ^b^	33.4 ± 4.4 ^b^	35.3 ± 1.8 ^b^

Values are expressed as the mean ± SEM (*n* = 8). One-way analysis of variance (ANOVA) followed by a Bonferroni post hoc. *p* < 0.05 was considered significant. The different letters represent significant differences.

**Table 2:** Sequence of primers used for RT-qPCR.

Gene	Forward Primer Sequence	Reverse Primer Sequence
*Il6*	CCTGGAGTTTGTGAAGAACAACT	GGAAGTTGGGGTAGGAAGGA
*Il1b*	TCAAGCAGAGCACAGACCTG	ACTGCCCATTCTCGACAAGG
*Il18*	TCGAAGCTTCCAAATCACTTC	TGAAGTTGACACAAGAGCCTTC
*Tnf*	TGAACTTCGGGGTGATCG	GGGCTTGTCACTCGAGTTTT
*Lep*	GGTGGCTGGTTTGTTTCTGT	TATGTGGCTGCAGAGGTGAG
*Il4*	TCCTTACGGCAACAAGGAAC	GTGAGTTCAGACCGCTGACA
*Adipoq*	TGGTCACAATGGGATACCG	CCCTTAGGACCAAGAACACCT
*Socs1*	GTCGGAGGGAGTGGGTGT	CGAGAGGCGGGATAAGGT
*Socs3*	CGGAACCTTCCTTTGAGGT	TGTAGTAAGCTCTCTTGGGGGTA
*Stat3*	CCTTGGATTGAGAGCCAAGAT	ACCAGAGTGGCGTGTGACT
*Pparg*	GGGGGTGATATGTTTGAACTTG	CAGGAAAGACAACAGACAAATCA
*Hif1a*	AAGCACTAGACAAAGCTCACCTG	TTGACCATATCGCTGTCCAC
*Nfkb1*	TCATCAACATGAGAAACGATCTG	CTCAGCAAGTCCTCCACCA
*Lipe*	TGAGAATGCCGAGGCTGT	AATTACCACATGGGAAGAAAGG
*Β-actin*	CTAAGGCCAACCGTGAAAAG	TACATGGCTGGGGTGTTGA

All primers were designed for a Tm of 60 °C.

## Data Availability

Not applicable.

## Supplementary Materials

The following supporting information can be downloaded at: https://www.mdpi.com/article/10.3390/molecules28093801/s1, Methodology and results of UPLC analysis. Table S1: Characterization by Ultra Performance Liquid Chromatography mass spectrometry, Table S2: Composition of (A) standard and (B) high-fat diets, Table S3: Differences in fold-change of all significant results of the treatment groups against the obesity control group.
