# Supplementary material for: Anti-Inflammatory Effect of Ethanolic Extract from Tabebuia rosea (Bertol.) DC., Quercetin, and Anti-Obesity Drugs in Adipose Tissue in Wistar Rats with Diet-Induced Obesity
Source: Molecules. 2023 Apr 28;28(9):3801. doi: 10.3390/molecules28093801 (PMC10180162; doi:10.3390/molecules28093801)
Supplement: Supplementary file 1 [file molecules-28-03801-s001.zip › molecules-2318485-Supplementary.pdf]

Characterization by Ultra Performance Liquid Chromatography mass spectrometry

The mass spectrometry analysis was carried out in an Acquity Ultra Performance Liquid Chromatography I-class system with a diode array detector (Waters Corp. Milford, MA, USA) coupled to a mass spectrometer with an ESI ionization source and time of flight VION IMS (Waters Corp. Milford, MA, USA).

The conditions of the analysis were the following:
The sample was diluted in mass grade methanol to a concentration of 2.5 mg/mL. The column used was BEH C18 2.1 x 100 mm, 1.7 µm. The mobile phases used were acetonitrile and water, both acidified with 0.1% formic acid.

The gradient used was the following:

Time	Phase A	Phase B
0	95	5
2	95	5
22	5	95
25	5	95
27	95	5
30	95	5

with a flow rate of 0.4 mL/min, column temperature 35°C, sample temperature 10°C, injection volume 2 µL. An absorbance scan was carried out from 210 to 600 nm, with specific channels: 214, 280, 320, 360 (and 520 nm in positive mode). Ionization was carried out in positive and negative mode. The mode of analysis was MSE, in which the low collision energy was 6 eV, with a ramp from 15 to 45 eV at high energy. The range of masses was considered from 50 to 1800 m/z. The capillary voltage

used was 2 and 3.5 kV for negative and positive respectively, source temperature 120 °C, desolvation temperature 450 °C, Argon as collision gas and desolvation with a flow of 50 L/h and 800 L/h. , respectively, and a cone voltage of 40 V. Leucine Enkephalin was used at a concentration of 200 pg/µl as reference for mass correction with a flow of 10 µL/min.

For data analysis, UNIFI, version 1.9 SR4 Software (Waters Corp., Milford, MA, USA) was used with the libraries of the Specialized Food Analysis Laboratory, The University of Mississippi Botanical Library and The University of Ottawa Phytochemical Library. Target match tolerance set to 5 ppm. For the identification of fragments, it was compared with fragmentation patterns reported in PubChem, FooDB version 1.0, HMDB version 5.0.

Table S1. Characterization by Ultra Performance Liquid Chromatography mass spectrometry

Compound	Response	Tr (min)	Molecular Formula	Expected Mass (g/mol)	Observed Mass (g/mol)	Fragment ions (negative mode) (m/z)								
Ursolic acid	498472	18.07	C30H4803	456.70	456.36	455.35	456.36	457.36						
Eriodictyol	288650	7.09	C15H12O6	288.25	288.06	125.02	151.00	124.02						
Fisetin	217868	9.22	C15H10O6	288.24	286.05	161.03								
Chamaemeloside	187439	6.98	C27H28O14	576.50	576.15	145.03	345.10	231.07	119.05	163.04	117.03	327.09	111.01	461.15
Salvigenin	178784	8.73	C18H16O6	328.30	328.10	327.09	297.08	311.06						
Quercetin	160013	8.21	C25H1007	302.23	302.04	301.04	151.00	179.00	121.03	107.02				
Naringenin	154218	9.03	C15H1205	272.25	272.07	119.05	151.00	177.02	107.01					
7-hydroxy-2-(1'hydroxyethyl)naphto[2,3-b]furan-4,9-dione)	154218	9.03	C15H12O5	272.25	272.07	151.00	107.01	93.04						
20R Ginsenoside	110218	21.10	C42H72013	785.00	784.49	737.48	475.31	367.30	460.28					
Harpagoside	106377	7.60	C24H30O11	494.50	494.18	345.10	145.03	433.15	119.05	231.07	337.11	391.14	475.16	
Khellin	102299	7.09	C14H1205	260.24	260.07	243.07	177.06	241.05	203.04					
Luteolin	59655	6.25	C15H1006	286.24	286.05	133.03	175.04	151.04	107.05					
Taxifolin	52409	6.25	C15H1207	204.25	304.06	285.04	125.02	301.04	273.04	227.07				

Ricinoleic acid	42577	15.58	C18H3403	298.50	298.25													
m-Coumaric acid	35295	5.59	C15H18O8	326.30	164.05	119.05	117.03											
Quercetin-7-O-glucoside	34257	7.30	C9H8O3	164.16	464.10	301.04	463.09	283.10	161.02									
Brachyposide A	32852	6.43	C29H36O15	624.60	624.20	307.08	461.17	271.06	445.15	593.19	607.20	339.05	235.06	433.15	505.17	551.18	207.07	167.03
Multifidoside B	32852	6.43	C29H36O15	624.60	624.20	307.08	461.16	271.06	445.15	339.05	235.06	207.07	167.03					
Betulonic acid	32631	19.32	C30H446O3	454.70	454.34	325.18												
D-(+)-Mannose	30725	0.63	C6H12O6	180.16	180.06	89.02	113.02	161.05	119.04	101.02	59.01	143.03						
Nipponogenin E	30328	13.27	C30H4805	488.70	488.35	469.33	249.22	293.21	429.30									
Chrysosplenetin	23453	10.39	C19H18O8	3744.30	374.10	145.03	299.06	343.08	193.01									
Rhein	20936	9.22	C15H8O6	284.22	284.03	239.04	163.00											
Verbascoside	17757	6.71	C29H26O15	624.60	624.20	623.20	179.04	461.17	624.20									
Hesperetin	17043	9.23	C16H14O6	302.28	302.08	301.07	286.04	257.04										
Isorhamnetin	14272	9.42	C26H12O7	316.26	316.06	300.03	315.05	301.03										
Luteolin-7-O-b-D-glucoside	13118	6.91	C21H20O11	448.40	448.10	285.04	307.08	339.05	133.03									
Scutellarein	12716	8.19	C15H10O6	286.24	286.05	117.04	285.04	255.03										
Maslinic acid	10844	21.95	C30H48O4	472.70	472.36	409.35	393.31											
Hesperidin	9702	7.11	C28H34O15	610.60	610.19	259.06	287.06	125.02	177.06	447.13	361.09	124.02						
trans-Piceatannol	8913	7.09	C14H12O4	244.24	244.07	243.07	201.06	215.07	173.06	225.06								
Linoleic acid	8791	19.60	C18H32O2	280.40	280.24	249.22												
Enhydrin	7908	7.61	C23H28O10	464.50	464.17	345.20	433.15	231.07	391.14									
Glaucolide A	7908	7.61	C23H28O10	464.50	464.17	345.10	433.15	231.07	391.14	175.04								
Methyl vanillate	7759	10.59	C9H10O4	182.17	182.06													
Eupatoriopicrin	6704	11.99	C20H26O6	362.40	362.17	221.15	220.15	236.11	205.12	148.05	177.09	293.18						
Enhydrin	6135	7.86	C23H28O10	464.50	464.17	345.10	433.15	231.07	307.08	301.04								
Betulonic acid	5018	19.62	C30H46O3	454.70	454.34	339.20												
Terminolic acid	4816	13.92	C30H48O6	504.70	504.34	457.33	413.34	309.21										
Esculetin	4672	6.25	C9H6O4	178.14	178.03	161.02	163.04	125.02	137.02									
Epicatechin	4540	7.25	C15H1406	290.27	290.08	289.07	137.02	125.02										
Linolenic acid	4146	18.43	C18H30O2	278.40	278.22													

Ciwujiatone	4052	7.51	C22H26O9	434.40	434.16	249.08	181.05	161.02	135.05	221.08	261.08	123.05					
Veratric acid	4020	7.50	C9H10O4	182.17	182.06	135.05	137.06	151.04	133.03	150.03	123.05	122.04	107.05				
Procyanidin B3	3974	8.73	C30H26O12	578.50	578.14	327.09	285.04										
Epiyangambin	3504	10.56	C24H30O8	446.50	446.19	211.10											
Apigenin	3124	7.09	C15H10O5	270.24	270.05	151.00	149.06	269.05	107.01								
Conicaoside	2628	5.97	C27H36O12	552.60	552.22	341.11	361.17	419.17	164.05	179.07	373.13	421.19	195.07	329.14			
Elemicin	2092	10.75	C12H16O3	208.25	208.11	135.05											
Cnicin	1955	16.54	C20H26O7	378.40	378.17	309.17											
Acanthosessiligenin I	1808	19.32	C31H48O5	500.70	500.35	325.18	453.34										
Epiaschantin	1646	11.68	C22H24O7	400.40	400.15												
Cubebin	1628	8.70	C20H20O6	356.40	356.13	175.04											
Conicaoside	1570	9.55	C27H36O12	552.60	552.22	145.03	345.10	435.17	163.04	507.15	433.15	535.18	419.17	117.04	231.07	117.04	175.04
Flavokawain B	1501	8.74	C17H16O4	284.31	284.11	175.04	267.07	251.06									
Chiisanogenin	1480	13.79	C30H44O5	484.70	484.32												
5-hydroxy-2-(1'hydroxyethyl)naphto[2,3-b]furan-4,9-dione)	1412	9.39	C14H10O5	258.23	258.05	241.01											
Arjungenin	1410	10.85	C30H48O6	504.70	504.35	193.09	133.03										
Eleutheroside E1	1388	6.90	C28H36O13	580.60	580.21	307.08	523.14	175.04	389.12								
Bakkenolide C	1387	11.48	C20H28O5	348.40	348.19	145.03											
Caffeic acid 3-glucoside	125	4.32	C15H18O9	342.30	342.09	179.04	161.04	149.05	89.03	165.06	123.01	327.11	138.03				

**Compound**	**Response**	**Tr (min)**	**Molecular Formula**	**Expected Mass (g/mol)**	**Observed Mass (g/mol)**	**Fragment ions (positive mode) (m/z)**							
Coumarin	57245	7.00	C9H6O2	146.14	146.04	147.04	119.05	91.05	148.05				
Specioside	44496	7.00	C24H28O12	508.50	508.16	147.04	119.05	321.13	165.06	329.10	369.10	311.09	183.07

Cedrelanol	20725	20.90	C15H26O	222.37	222.20	205.20	93.07	135.12	91.05	121.10	95.09	149.13
Salvigenin	20502	7.00	C18H16O6	328.30	328.09	311.09	147.04	119.05				
Luteolin	19955	9.24	C15H10O6	286.24	286.05	287.06	153.02					
Morin	14519	8.24	C15H10O7	302.23	302.04	147.05	303.05	229.05				
Caryophyllene	14432	20.90	C15H24	204.35	204.19	93.07	135.12	91.05	121.10	95.09	149.13	
Acanthopanaxoside E	12597	13.66	C42H66O15	811.00	810.44	189.10	614.35	547.34	279.13	476.31		
Germbudine	10240	10.87	C37H59NO12	709.90	709.40	519.32						
Germine	10003	7.97	C27H43NO8	509.60	509.30	195.07	479.28					
Gigantecin	9419	22.05	C37H66O8	638.90	638.48	541.38	325.24	497.35	297.25	205.20		
Eleutheroside B1	9301	0.95	C17H20O10	384.30	384.11	209.04						
Eriodictyol	9231	8.11	C15H12O6	288.25	288.06	163.04	153.02					
Cinerenin	5063	6.94	C17H20O6	320.30	320.12							
Homovanilic Acid	4716	7.53	C9H10O4	182.17	182.06	165.05	137.06					
Acanthosessilioside C	4540	11.61	C37H58O12	694.80	695.40	471.29	447.20					
Acantrifoic acid A	4034	17.85	C32H48O7	544.70	530.33	89.06	503.31					
Galphin A	2826	14.91	C34H48O10	616.70	617.33	189.10	569.32	481.26	437.24	247.10		
Cubebin	147	11.01	C20H20O6	356.40	356.13	137.06						

Table S2. Composition of (A) standard and (B) high-fat diet.

A)

Component	Composition g/100g diet
Protein	23.9
Fat	5.7
Fiber crude	5.1
Carbohydrates*	38.13
Minerals	7%
Vitamin mix	#

* According to the compounds that are carbohydrates in the formulation

B)

Component	Composition g/100g diet
Sucrose	34.0
Milk fat	21.0
Casein	19.5
Corn starch	14.05
Cellulose	5.0
Mineral mix [1]	4.3
Vitamin mix [2]	1.5
DL methionine	0.3
Choline	0.2
Cholesterol	0.15

[1]Mineral mix (AIN-93 G-MX); [2]Vitamin mix (AIN-93-VX)

Table S3. Differences in fold-change of all significant results of the treatment groups against the obesity control group.

Log2 Fold-change VS HF-Ob

	HF+Tr	HF+Q	HF+Orl	HF+F	HF+P
**IL6**	-3.67173522	-5.13495448	-6.26428298	-3.77567608	-3.7154972
**IL4**	1.72406245	0.29591517	2.16336426	-1.67851493	0.73740848
**IL-1B**	-1.50404251	-1.33536713	0.47424823	-1.30159425	-0.29024078
**IL18**	-0.64711898	-1.36448335	-0.28228229	-1.07272038	-0.89283651
**TNF**	0.63226163	0.87073611	1.51150947	1.03706135	0.80335623
**LEP**	-0.82198346	-2.70148701	1.18522103	1.26622268	0.11339368
**ADIPONECTIN**	0.9156736	0.85779269	-0.0529448	0.07594325	0.7293004
**SOCS1**	0.31826856	1.26829312	6.41180034	5.91706202	6.95258624
**SOCS3**	0.92111623	0.89325113	4.54117643	2.55473454	4.07786973
**STAT3**	-0.10643186	0.2410081	-0.39711559	-0.5320487	-1.09573487
**NF-KB**	-2.24023919	-2.23603767	-4.4243291	-5.09002545	-3.68163852
**PPARG**	-0.89695562	-0.70984617	-2.29721776	-1.90031875	-0.91116948
**HIF-IA**	-1.31866408	-1.38539019	-2.81128152	-3.24362464	-2.40667466
**LIPE**	1.23874621	-1.09108011	-3.15988633	-1.54732176	-4.63191328

The negative sign indicates under-expression compared to the obese control group (HF-Ob), while the positive sign indicates mRNA overexpression.
